# Effect of Hybrid Carbon Fillers on the Electrical and Morphological Properties of Polystyrene Nanocomposites in Microinjection Molding

**DOI:** 10.3390/nano8100779

**Published:** 2018-09-30

**Authors:** Shengtai Zhou, Andrew N. Hrymak, Musa R. Kamal

**Affiliations:** 1 Department of Chemical and Biochemical Engineering, The University of Western Ontario, London, ON N6A 5B9, Canada; szhou96@uwo.ca; 2 Department of Chemical Engineering, McGill University, Montreal, QC H3A 0C5, Canada; musa.kamal@mcgill.ca

**Keywords:** microinjection molding, hybrid fillers, multi-walled carbon nanotubes, carbon black, conductive polymer composites, microstructure

## Abstract

The effect of hybrid carbon fillers of multi-walled carbon nanotubes (CNT) and carbon black (CB) on the electrical and morphological properties of polystyrene (PS) nanocomposites were systematically investigated in microinjection molding (μIM). The polymer nanocomposites with three different filler concentrations (i.e., 3, 5 and 10 wt %) at various weight ratios of CNT/CB (100/0, 30/70, 50/50, 70/30, 0/100) were prepared by melt blending, then followed by μIM under a defined set of processing conditions. A rectangular mold insert which has three consecutive zones with decreasing thickness along the flow direction was adopted to study abrupt changes in mold geometry on the properties of resultant microparts. The distribution of carbon fillers within microparts was observed by scanning electron microscopy, which was correlated with electrical conductivity measurements. Results indicated that there is a flow-induced orientation of incorporated carbon fillers and this orientation increased with increasing shearing effect along the flow direction. High structure CB is found to be more effective than CNT in terms of enhancing the electrical conductivity, which was attributed to the good dispersion of CB in PS and their ability to form conductive networks via self-assembly. Morphology observations indicated that there is a shear-induced depletion of CB particles in the shear layer, which is due to the marked difference of shear rates between the shear and core layers of the molded microparts. Moreover, an annealing treatment is beneficial to enhance the electrical conductivity of CNT-containing microparts.

## 1. Introduction

Traditionally, conductive polymer composites (CPCs) demonstrate potential applications in the areas of antistatic, electromagnetic interference shielding, thermal management, fuel cells, sensing and so forth [1,2,3,4,5,6]. The CPCs can be prepared by melt blending which is compatible with current industrial processing techniques, such as extrusion and/or injection molding [7]. To make a polymer composite conductive, it is paramount for electrically conductive fillers to attain a three-dimensional (3D) network within the host polymer matrix. The critical filler concentration at which the polymer matrix translates from an insulator to a conductor is recognized as the percolation threshold (*p*_c_) [8]. Multi-walled carbon nanotubes (CNT), which are characteristic of high aspect (length to diameter) ratio [9], intrinsically high thermal and electrical conductivity [10] have been extensively adopted as one of the most important functional fillers to fabricate CPCs. However, commercial CNT is supplied in the form of heavily entangled bundles, which makes it difficult to disperse in a polymer matrix [10].

To achieve higher electrical conductivity, the concept of hybrid fillers, i.e., the utilization of fillers with different morphologies or aspect ratios has been adopted by researchers to prepare CPCs. Zhang et al. [1] revealed that the *p_c_* of polypropylene (PP) composites loaded with hybrid fillers of CNT and carbon black (CB) is much lower than that of only CNT or CB-containing counterparts. For example, they found that the *p*_c_ can be reduced from 2.4 to 0.21 wt % by simply substituting half of the CNT with CB [1]. Shen and coworkers [11] reported that the volume electrical conductivity of high density PE incorporated with hybrid fillers of CB and carbon fiber is nearly three orders of magnitude higher than that of only CB-containing counterparts, at the same total filler concentrations. In addition, Liang et al. [12] reported that the addition of silver particles into PP is advantageous to the formation of conductive pathways in the presence of CNT, leading to a significant increase of electrical conductivity.

Recently, there has been increasing demand for micro-components in the areas of electronics, automotive, microelectromechanical systems (MEMS) and microsystems [13,14]. Microinjection molding (μIM) is becoming an important technology thanks to its suitability for mass production of microparts with complex shape and high surface quality at relatively low cost [13]. So far, a number of studies have been conducted with respect to the μIM of unfilled thermoplastics [15,16,17,18]. However, μIM of filler-containing polymer composites is receiving attention due to their potential multifunctional performance. For instance, Abbasi et al. [18] studied the properties of microinjection molded polycarbonate (PC)/CNT and PP/CNT nanocomposites. The authors of [18] found that the *p*_c_ for both CNT-containing microparts shifted to higher filler concentrations when compared with their compression molded counterparts, which is attributed to the prevailing high shear rates in μIM. Similar findings were reported by Zhou et al. [19] in different types of carbon fillers loaded PP nanocomposites in µIM. Additionally, they found [19] that the CNT and high structure CB are more effective than graphite nanoplatelets (GNP) in enhancing the electrical conductivity of PP-based nanocomposites, which is closely related to the development of microstructure in corresponding microparts. For instance, a “grape-like” structure was typically observed in PP/CB microparts, and a conductive network could be formed in CNT-containing counterparts thanks to its higher aspect ratio. However, such morphology was hardly detected in either the compression molded PP/GNP composites or their microparts. Nevertheless, the presence of crystal structures in PP could affect the distribution of incorporated fillers [18]. The crystal structures promote a morphology similar to that of a double-phase immiscible polymer blend, which might lead to the aggregation of nano-fillers in the less crystalline or amorphous regions [18]. Therefore, an amorphous polymer, polystyrene (PS), was employed as the host polymer to minimize the above-stated influence. In addition, PS has been widely adopted as the polymer matrix for hosting hybrid fillers [20,21,22]. CNT and CB were adopted as conductive fillers. To the best of our knowledge, there is little research available with respect to the μIM of hybrid fillers modified CPCs.

In present study, a series of PS nanocomposites modified with hybrid fillers of CNT and CB at various weight ratios (100/0, 30/70, 50/50, 70/30 and 0/100) were fabricated by melt blending. The objective of this study is to explore the effect of hybrid carbon fillers on the electrical and morphological properties in μIM. To this end, a rectangular mold insert which has three consecutive zones with decreasing thickness along the flow direction was utilized to prepare the microparts [23]. As a result, the effect of abrupt changes in mold cavity thickness on the electrical and morphological properties of corresponding microparts was detailed. 

## 2. Materials and Methods

### 2.1. Materials

**Figure 1:**
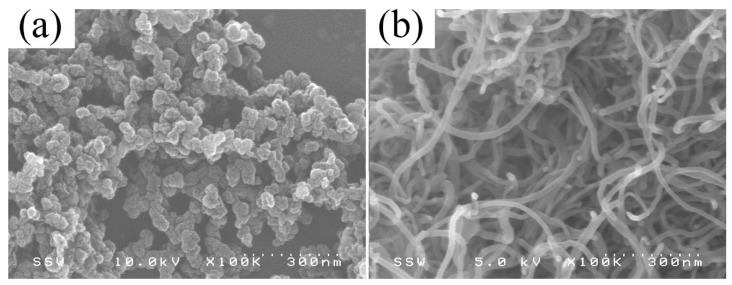
The morphology of (**a**) high structure carbon black and (**b**) multi-walled carbon nanotubes.

Polystyrene (PS, Grade: GPPS 1540) was obtained from Tabriz Petrochemical Company (Tabriz, Iran). The polymer has a density of 1.04 g/cm^3^ and a melt flow index of 11 g/10 min (200 °C/5 kg). The carbon black (CB, Tradename: Ketjenblack^®^ EC-600JD) was supplied by Akzo Nobel Polymer Chemicals LLC (Chicago, IL, USA). The highly branched CB has an electrical conductivity of 0.01–0.1 Ω cm, a density of 1.80 g/cm^3^ and a pore volume of 480–510 cm^3^/100 g [24]. The CB has a very large surface area, which is about 1400 m^2^/g [19]. The mean particle size of CB is determined in a range from 30 to 50 nm. The primary CB particles have a high tendency to form a chain-like structure which is crucial to the transport of electrons, as shown in Figure 1a. An industrial grade of multi-walled carbon nanotubes (CNT, Grade: TNIM2) was purchased from Chengdu Organic Chemicals Co., Ltd. (Chengdu, China). The CNT was prepared by chemical vapor deposition method with a length of 30–50 µm. The surface area for CNT is 230 m^2^/g. According to supplier, the outer and inner diameter of CNT are 8–15 and 3–6 nm, respectively. The morphology of highly entangled CNT agglomerates is given in Figure 1b.

### 2.2. Preparation of Microparts

**Figure 2:**
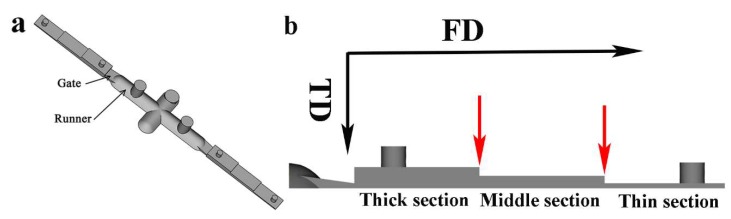
(**a**) 3D view of a final micropart; (**b**) three step decrease configuration of the micropart, the arrows indicate the boundary of each section.

A Brabender internal batch mixer (C.W. Brabender Instruments, South Hackensack, NJ, USA), equipped with two counter-rotating blades, was used to prepare different carbon fillers loaded polymer composites. According to a previous study [25], the *p*_c_ for PS/CNT microparts ranges from 5–7 wt %. Thus, the concentration of total carbon fillers in PS was selected at 3, 5 and 10 wt %, respectively. However, the weight ratio of CNT to CB was systematically altered at various combinations (i.e., 100/0, 30/70, 50/50, 70/30 and 0/100). The obtained samples were named as *x*-PS/CNT*y*0/CB*z*0, where *x* is the weight fraction of total carbon fillers, *y* and *z* are the weight ratio of CNT and CB in the total carbon fillers. To put it simply, 3 wt %-PS/CNT30/CB70 represents the sample with 3 wt % total carbon fillers and the weight ratio of CNT to CB is 3:7, i.e., PS(97 wt %)/CNT(0.9 wt %)/CB(2.1 wt %). The nomenclature also applies to other carbon filled systems. The compounding process was carried out at 200 °C and 50 rpm for 10 min. The obtained composite was mechanically crushed and used for µIM. The micromolding machine, Battenfeld Microsystem 50 (Wittmann Battenfeld GmbH, Kottingbrunn, Lower Austria, Austria), features a plunger injection system, which consists of a screw melting unit, a metering unit and an injection unit [13]. The melt temperature and mold temperature were 260 and 80 °C, respectively. The injection speed was 100 mm/s. Figure 2a displays 3D schematic view of a final micropart. All sections of the micropart have a same width of 2.40 mm and the thickness of thick, middle and thin sections is 0.85, 0.50 and 0.20 mm, respectively. The thick and middle sections have a length of 5.00 mm while the thin section has a length of 4.80 mm.

### 2.3. Characterizations

#### 2.3.1. Electrical Conductivity

Samples for electrical conductivity measurements were cut into three sections from microparts using a scalpel, which is shown in Figure 2b, as indicated by the red arrows. Direct current (DC) electrical conductivity was measured by a two-probe method [26] for each section of the microparts in two directions, i.e., parallel (FD) and perpendicular (TD) to the predominant melt flow direction. The resistance (*R*, Ω) of each sample was determined by a Keithley 6514 electrometer (Tektronix, Inc., Beaverton, OR, USA). According to the manual for Keithley 6514, the unit can make measurements from 10 mΩ to 210 GΩ. Thus, the lower limit for the Keithley 6514 is 210 GΩ where no effective current signal can be detected. Afterwards, the obtained *R* was converted into volume electrical conductivity (σ, S/cm) using the following equation:

(1) $$\;\sigma =\frac{1}{\rho }=\frac{L}{AR}\;$$

where ρ is the volume electrical resistivity, *L* (cm) is the distance between the copper electrodes and *A* (cm^2^) is the surface contact area. Five specimens were tested for each measurement.

#### 2.3.2. Morphology

The samples used for morphology observations were cryogenically fractured across the TD in liquid nitrogen, followed by coating a thin layer of platinum to enhance surface conductivity. A high-resolution scanning electron microscope (SEM, Hitachi S-4500, Tokyo, Japan) with an acceleration voltage of 5 kV was employed to probe the microstructure across the cross-section of the microparts.

## 3. Results and Discussion

### 3.1. Electrical Conductivity

**Figure 3:**
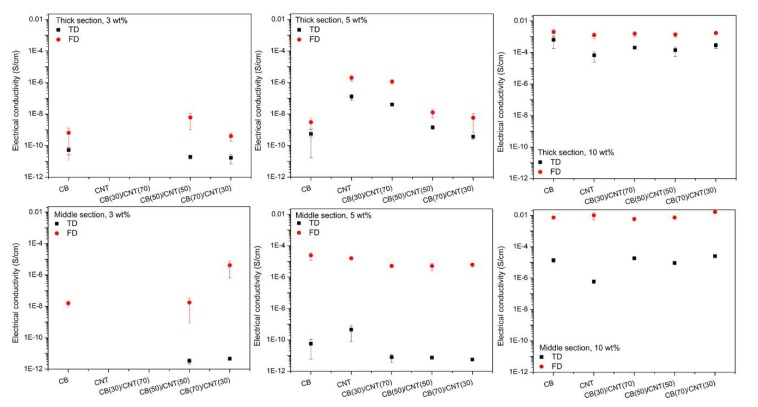
The σ for both the thick and middle sections of microparts which were molded from different carbon filled PS nanocomposites. The measurements were conducted with respect to the TD and FD, respectively.

The DC electrical conductivity (σ) for both the thick and middle sections of microparts with respect to measurement directions is reported in Figure 3, where is shown that FD σ for either section of the microparts is higher than that measured across the TD. This behavior is indicative of preferred orientation of carbon fillers along FD, arising from the dominant shearing effect that prevails in the injection molding process [27]. Furthermore, it should be kept in mind that in addition to the stepped decrease in thickness of the mold cavities along FD [25], the adopted molding parameters, such as melt and mold temperatures as well as injection speed, are basically higher than those encountered in conventional injection molding (CIM) [19,28]. As a result, the shear rate generated in μIM is at least two orders of magnitude higher than that in CIM [29], which is accountable for the preferential orientation of added fillers in the microparts [30]. For instance, Abbasi et al. [30] found that the *p*_c_ for PC/CNT microparts shifted to higher filler concentrations when compared with that of compression molding or CIM counterparts. Meanwhile, the preferred orientation of carbon fillers along the FD would be detrimental to the random formation of conductive pathways across the TD within the host matrix [19]. Therefore, the TD σ for the middle section is lower than their thick section counterpart, at a specified filler concentration. For instance, the TD σ for middle section of PS/CB 3 wt % microparts is beyond the lower limit of the measurement scale for Keithley 6514 electrometer whereas the TD σ for their thick section counterparts is about 5.12 × 10^−11^ S/cm.

In addition, it is worth mentioning that the σ for either the thick or middle section of PS/CNT 3 wt % microparts (regardless of the measurement directions) is beyond the lower limit of the Keithley electrometer, indicating a lack of conductive pathways. This finding is consistent with the results reported by Arjmand et al. [31] where the authors found that the *p*_c_ for PS/CNT nanocomposites is about 5 wt % in CIM. Thus, the prevailing shearing effect in μIM would largely limit the possibility of CNT-CNT contacts in the microparts [30]. However, the values of σ for both sections of PS/CB 3 wt % microparts are measurable except that the TD σ for the middle section is out of the lower limit for the Keithley 6514 electrometer. The above description indicated that despite the intrinsically high aspect ratio and electrical conductivity of nanotubes [32], the state of dispersion of the added fillers plays a significant role in determining the σ of subsequent polymer nanocomposites [33]. Additionally, the ratio of FD σ to TD σ, i.e., (FD/TD)_σ_, for the middle section is invariably higher than that of the thick section (see Table 1), confirming that the increasing shearing effect would facilitate filler orientation along the predominant flow direction. Therefore, the values of (FD/TD)_σ_ can be used as an indicator to assess the degree of filler orientation in the injection molded CPCs. 

Moreover, the values of (FD/TD)_σ_ decrease with an incremental loading concentration of added fillers, indicating that the difference between the TD σ and FD σ minimized with increasing carbon filler concentrations. In this case, it showed that there is a higher probability to form 3D conductive pathways within subsequent moldings at higher filler loading fractions, regardless of shear-induced orientation of incorporated fillers along the flow direction. Besides, the trend of decreasing ratio of (FD/TD)_σ_ with an increase of filler concentration is applicable to either only CNT or hybrid carbon fillers (regardless of the weight ratio of CNT/CB) loaded PS microparts.

Similar to PS/CNT 3 wt % microparts, the values of σ for both the thick and middle sections of 3 wt %-PS/CNT70/CB30 microparts cannot be determined using the Keithley 6514 electrometer due to insufficient conductive pathways; however, corresponding values could be detected for the other combinations of hybrid carbon fillers loaded PS systems (at the same total filler concentration, i.e., 3 wt %) where CB occupies a relatively higher weight fraction, suggesting that high structure CB is more effective than CNT in enhancing the σ of subsequent moldings. In this scenario, the polymer-filler interfacial interaction might be an influencing factor. According to Clingerman et al. [34], a lower interfacial tension between polymer matrix and fillers promotes a better wettability of polymer chains with the added fillers, thereby leading to an improved dispersion of added fillers in the host matrix. As a result, the improved dispersion of conductive fillers would, in turn, increase the *p_c_* of CPCs [35].

The values of surface tension for PS, CB and CNT, and the interfacial tension between PS and different carbon fillers at 260 °C are listed in Table 2. The interfacial tension between polymer/filler pair was calculated using the Wu’s harmonic mean average equation [36].

(2) $$\;\gamma_{12}=\gamma_{1}+\gamma_{2}-4\left(\frac{\gamma_{1}^{d}\gamma_{2}^{d}}{\gamma_{1}^{d}+\gamma_{2}^{d}}-\frac{\gamma_{1}^{p}\gamma_{2}^{p}}{\gamma_{1}^{p}+\gamma_{2}^{p}}\right)\;$$

where $\gamma_{12}$ is the interfacial tension between component 1 and component 2; $\gamma_{i}$ is the surface tension of component *i*, which equals to $\gamma_{i}^{d}$ and $\gamma_{i}^{p}$. In addition, $\gamma_{i}^{d}$ and $\gamma_{i}^{p}$ are the dispersion part and the polar part of surface tension of the *i*^th^ component, respectively.

Table 2 reveals that the interfacial tension of PS/CB pair is lower than that of the PS/CNT pair, indicating that polymer chains could easily wet outer surface of CB particles, which leads to an improved dispersion of CB in PS. The improved dispersion of CB particles explains the fact that although a direct contact of conductive fillers is unlikely at a lower total filler concentration (i.e., 3 wt %) [31], it allows free passage of electrons though the ‘tunneling’ or ‘hopping’ mechanism in the host matrix [39]. It is thus not surprising that the values of σ for both the thick and middle sections of microparts molded from 3 wt %-PS/CNT70/CB30 and PS/CNT 3 wt % nanocomposites are not detectable due to a lack of sufficient conductive pathways, regardless of the measurement directions. In addition, a typical synergistic effect of hybrid carbon fillers on the σ for both the thick and middle sections of 3 wt %-PS/CNT50/CB50 microparts is discernible since the values of σ for the 3 wt %-PS/CNT50/CB50 microparts are generally superior to those of PS/CNT 3 wt % or PS/CB 3 wt % counterparts. In this scenario, it could be deduced that the co-existence of CB particles with CNT facilitates the formation of conductive pathways within the host polymer.

When the total filler concentration is increased to 5 wt %, it is expected that enough conductive pathways can be constructed by CNT in PS because the values of σ for PS/CNT 5 wt % microparts is always higher than that of PS/CB 5 wt % counterparts, as shown in Figure 3. However, no synergistic effect of CNT and CB on the σ of subsequent microparts is detected since the σ for the thick section of hybrid filler-containing microparts is normally within the range of PS/CB 5 wt % (lower bound) and PS/CNT 5 wt % (upper bound) counterparts whereas the σ for the middle section is invariably lower than that of PS/CB 5 wt % or PS/CNT 5 wt % counterparts, regardless of measurement directions. Moreover, the σ for either section of PS/CNT/CB microparts increases with an incremental loading fraction of CNT, revealing that the presence of CNT is advantageous to the formation of conductive pathways. For example, the FD σ and TD σ for the thick section increase from 5.81 × 10^−9^ (5 wt %-PS/CNT30/CB70) to 1.16 × 10^−6^ S/cm (5 wt %-PS/CNT70/CB30) and from 3.66 × 10^−10^ (5 wt %-PS/CNT30/CB70) to 4.05 × 10^−8^ S/cm (5 wt %-PS/CNT70/CB30), respectively. A monotonic increase of FD σ for the thick section is attributed to the preferential alignment of CNT along FD since more conductive pathways can be constructed with an increasing weight fraction of nanotubes. Also, a concurrent increase of TD σ for the thick section is ascribed to the spatial dispersion of nanotubes across TD, thereby facilitating the formation of conductive pathways across TD, albeit the favored orientation of CNT along the flow direction. Furthermore, more filler orientation in the middle section is expected with an increase of shear rates, arising from the sharp decrease of the mold cavity thickness, from 0.85 (thick section) to 0.50 mm (middle section), along the predominant melt flow direction. Consequently, the FD σ for the middle section is always higher than that of its thick section counterpart whereas the TD σ for the thick section is higher than that of the middle section. Similarly, such trend also applies to microparts which have a total filler concentration of 10 wt %, in which case sufficient conductive pathways can be formed. In addition, Table 3 indicated that the presence of CB is generally beneficial to the enhancement of σ for subsequent microparts, which can be attributed to the uniform distribution of CB within the host matrix. 

Based on the range of total carbon filler concentrations studied, no obvious synergistic effect of hybrid loading of CNT and CB on the enhancement of σ for PS microparts was detected. According to a review by Szeluga et al. [40], hybrid carbon fillers loading of CNT and CB does not always guarantee a synergy in enhancing the σ of subsequent polymer composites. On the one hand, the ratio of carbon nanofillers in the hybrid mixture is crucial to determining the properties of polymer composites, such as the mechanical properties, thermal and electrical conductivity [40,41]. On the other hand, the interaction between polymer matrix and carbon nanofillers or the state of dispersion of conductive particles may play a role in constructing co-supportive conductive network since a synergistic enhancement of σ was typically observed for the semi-crystalline polymer, e.g., PP [1] and epoxy-based composites [32,42,43]. In a previous study [44], we have demonstrated that the intrinsic properties of polymer matrix would determine the state of dispersion of incorporated CNT, thereby affecting the σ of subsequent moldings. Thus, a thorough investigation on the influence of intrinsic properties of the host polymers on the properties of hybrid carbon fillers loaded systems would be helpful to elucidate this phenomenon.

Overall, the synergistic enhancement of σ could be achieved in PS microparts when the total carbon fillers concentration is 3 wt % wherein the CB and CNT have equivalent weight fractions, i.e., 3 wt %-PS/CNT50/CB50. Interestingly, the middle section of 3 wt %-PS/CNT30/CB70 microparts showed strong synergy in terms of the enhancement of σ when compared with other filler-containing counterparts. However, such effect was absent at higher filler concentrations and the σ of subsequent microparts tends to increase with increasing fractions of CNT and CB when the total filler concentration is 5 and 10 wt %, respectively.

**Figure 4:**
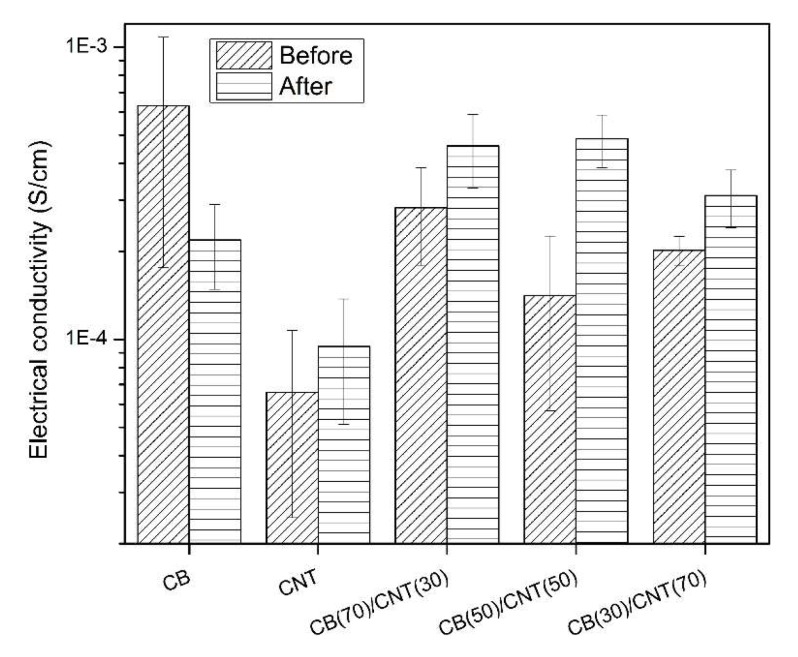
The TD σ for the thick section of different combinations of CB/CNT fillers loaded PS microparts. Results were collected from samples before and after the annealing treatment at 100 °C for 2 h.

To investigate the effect of annealing on the σ of micromoldings, microparts molded from the nanocomposites with 10 wt % total carbon fillers were subject to a thermal treatment at 100 °C for 2 h under vacuum. Figure 4 indicated that the TD σ for the thick section of PS/CB 10 wt % microparts decreased remarkably after the annealing treatment. Herein, two possible mechanisms are proposed to explain the annealing-induced decrease of σ for only CB-containing PS microparts: (1) the very high shear rate and large thermal gradients during μIM could largely induce a preferred orientation of polymer chains and the added fillers in flow direction. Thus, the rapid solidification of generated structure has little chance returning to a random orientation [44]. The mobility of polymer chains could be greatly improved during the annealing treatment, which leads to a relaxation of the orientated polymer chains to random coils [45]. In addition, since the adopted CB has a relatively higher surface area (~1400 m^2^/g) compared with the CNT, the dispersed CB particles have a greater tendency to form segregated agglomerates, which could disrupt the conductive pathways within the polymer matrix. (2) Since the mobility of the polymer chains can be greatly improved at higher temperatures, more macromolecular chains are likely to migrate to the outer surface of CB particles due to the good wettability of PS with CB. Moreover, the high surface area of CB could be a contributing factor because a certain amount of polymer chains is required to wet the surface of CB particles. A similar downward trend of σ with temperature was reported by Liang and Tjong [45] in a carbon nanofiber filled PS system. The authors [45] proposed that the increased mobility of polymer chains at higher temperatures would interrupt the random formation of conductive network, thereby increasing the resistivity of subsequent samples. Consequently, the annealing treatment is detrimental to the σ of only CB-containing samples.

Unlike PS/CB 10 wt % microparts, the TD σ for the thick section of only CNT or hybrid carbon fillers loaded PS counterparts increased after the annealing treatment. For example, the TD σ for the thick section of 10 wt %-PS/CNT50/CB50 microparts increased from 1.41 × 10^−4^ to 4.86 × 10^−4^ S/cm. The annealing-induced enhancement of σ can be explained as follows: as described previously, there would be a preferential alignment of carbon fillers along the flow direction arising from the predominant shearing effect and rapid solidification of as-molded products in μIM [8]. As a result, the residual stress and strain will exist in the interfacial polymer phases between the added fillers [46]. However, this is not a thermodynamically-favored state and, the frozen-in polymer chains and orientated structure would have a high tendency to reorder or rearrange themselves to a random orientation, provided that the polymer chains gain a certain degree of mobility. In addition, the presence of surrounding insulating polymers would increase the contact resistance and limit the possibility to construct conductive pathways through the direct contact of carbon fillers, thereby impairing the enhancement of σ. Li et al. [47] proposed that the annealing treatment could relieve the residual stress and strain that exists in the interfacial polymers between the added fillers thanks to the increased mobility of polymer chains. As a result, the mean distance between adjacent CNTs decreased slightly with an increase of annealing temperature [47], which in turn leads to a reduction of the ‘tunneling’ resistance, thereby contributing to the enhancement of σ. Moreover, the increased mobility of polymer chains promotes secondary agglomeration of CNT (e.g., loosely packed CNT network) [48,49], which is favorable for the enhancement of σ as well. Interestingly, a significant increase of the TD σ was observed for the thick section of 10 wt %-PS/CNT50/CB50 microparts, in which case the CB and CNT have equivalent weight fraction in PS, i.e., 5 wt % respectively. As mentioned previously, conductive pathways can be formed in either only CB- or CNT-containing PS microparts at 5 wt %. Thus, it is reasonable to suggest that the synergistic effect of hybrid carbon fillers on the increment of σ arises from the formation of CB aggregates and the restoration of loosely packed CNT network within the host matrix after the annealing treatment.

### 3.2. Morphology

**Figure 5:**
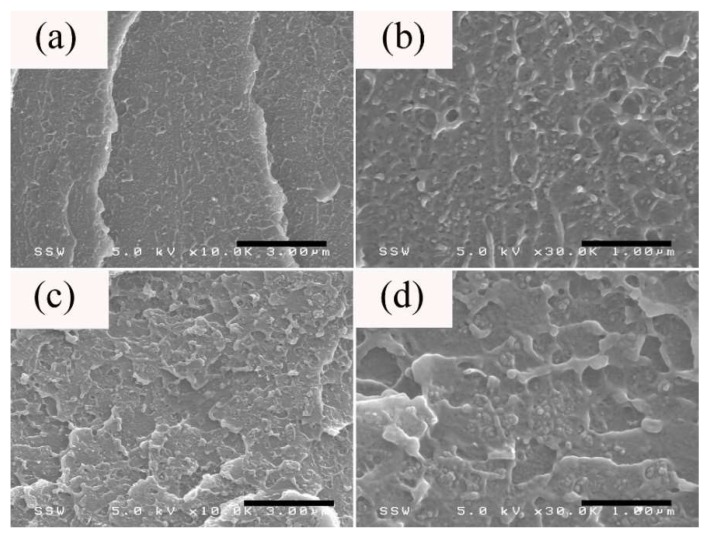
The morphology (TD) taken from the (**a**,**b**) core and (**c**,**d**) shear layers of the thick section of PS/CB 5 wt % microparts.

The cross-section morphology of the thick section of PS/CB 5 wt % microparts is shown in Figure 5. Figure 5a,b showed that the CB particles have a relatively uniform distribution in the core layer of the thick section, in a form of small aggregates or a chain-like structure, suggesting that the high structure CB particles could form a random conductive network via self-assembly even under very high shearing conditions, which is beneficial to the enhancement of σ. A similar phenomenon was observed by Yui and co-workers [50] in CIM PP/HDPE/CB macroparts. However, despite the uniform distribution of CB aggregates in the shear layer, there are some regions devoid of CB particles and the shear-induced depletion of CB particles could be the contributing factor. For example, Jana [51] reported that the loss of σ in CPCs could be attributed to shear-induced migration of conductive fillers during the mold filling process in injection molding. In addition, Hong et al. [52] reported that the migration of conductive fillers became more severe with an increase of shearing conditions. Jiang et al. [29] pointed out that shear rates as high as 10^6^/s are not rare in μIM, and the shear layer exhibits a higher shear rate relative to that of the core layer [25]. Therefore, the shear-induced migration of CB particles would become more significant in the shear layer, as shown in Figure 5d.

**Figure 6:**
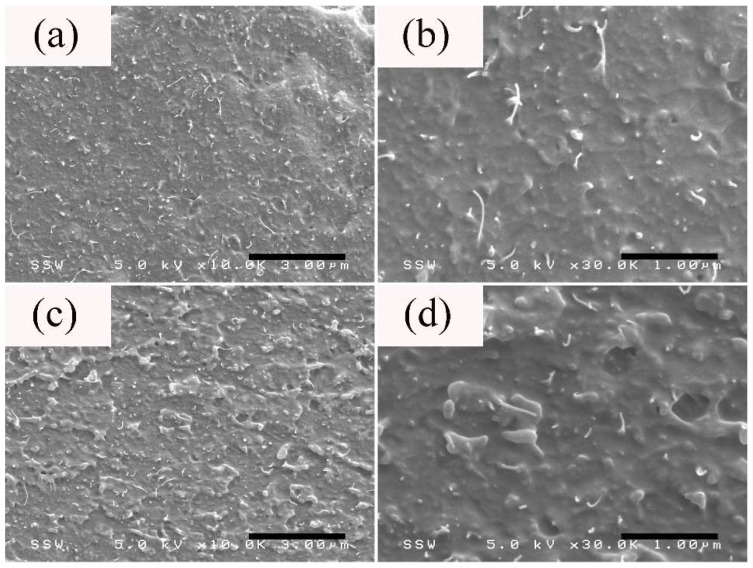
The morphology (TD) taken from the (**a**,**b**) core and (**c**,**d**) shear layers of the thick section of PS/CNT 5 wt % microparts.

The cross-section morphology of the thick section of PS/CNT 5 wt % microparts is shown in Figure 6. Figure 6 showed that CNT has a relatively uniform distribution across the TD. Moreover, CNT agglomerates seem to be absent from the thick section across the TD and no obvious shear-induced depletion effect on nanotubes-containing counterparts is observed in the shear layer. This suggests that there exists a good wettability between PS macromolecular chains and the outer surface of CNT, which leads to their good dispersion in the host matrix [25,53]. In addition, the particle size could play a role when it comes to the shear-induced migration phenomenon. For example, unlike CB particles, the adopted CNT has a very high aspect ratio (>1000). Thus, the shear-induced depletion effect might be insignificant in terms of the particle size of nanotubes and the rapid mold filling process in μIM.

**Figure 7:**
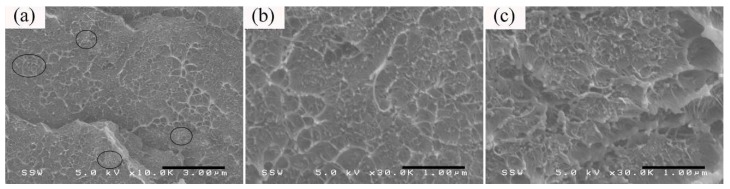
The morphology (TD) taken from the (**a**,**b**) core and (**c**) shear layers of the thick section of 5 wt %-PS/CNT50/CB50 microparts before the annealing treatment.

The cross-section microstructure for the thick section of 5 wt %-PS/CNT50/CB50 microparts, prior to annealing treatment, is displayed in Figure 7, indicating that both the CNT and CB have a relatively uniform distribution across the TD. The existence of CNT agglomerates could be detected in the core layer of microparts, which have been labeled in black contours, as shown in Figure 7a. However, the discretely dispersed CB and CNT would be unfavorable for the effective construction of conductive pathways within subsequent microparts. For example, Wu et al. [54] proposed that the co-existence of two independent phases, namely the particle phase (i.e., CB aggregates) and the bridge phase (i.e., individually dispersed CNT or CNT agglomerates) is unfavorable for the enhancement of σ. Consequently, the σ for 5 wt %-PS/CNT50/CB50 microparts is somewhat lower than that of only CNT-containing counterparts, as displayed in Figure 3.

**Figure 8:**
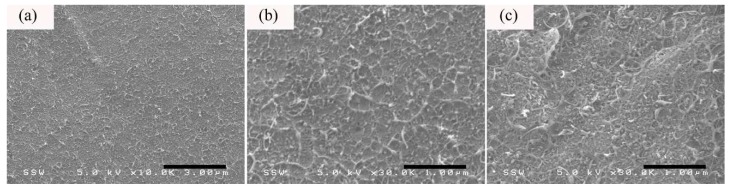
The morphology (TD) taken from the (**a**,**b**) core and (**c**) shear layers of the thick section of 5 wt %-PS/CNT50/CB50 microparts after the annealing treatment.

The cross-section microstructure for the thick section of 5 wt %-PS/CNT50/CB50 microparts after annealing treatment is given in Figure 8. Similar to Figure 7, both the CB and CNT have a relatively uniform distribution in PS. In addition, despite of the presence of individually dispersed CB and CNT, it seems that the annealing treatment would promote the secondary agglomeration of CNT [49,55] and the formation of CB aggregates in the host matrix [56], which are responsible for the enhancement of σ [57]. As a result, the TD σ for the thick section of 10 wt %-PS/CNT50/CB50 microparts is nearly 3.5 times higher than that obtained from their counterparts prior to the annealing treatment, as reported in Figure 4.

## 4. Conclusions

To conclude, a series of hybrid fillers of CNT and CB loaded PS nanocomposites were prepared by melt blending. The weight ratio of CNT/CB was systematically varied at the same filler concentrations. Afterwards, the obtained blends were subjected to μIM under a defined set of processing conditions. The distribution of carbon fillers within the micromoldings was evaluated by a combination of electrical conductivity measurements and morphology observations. Results indicated that both CB and CNT have a relatively uniform dispersion within PS. Although there is a shear-induced depletion effect in the shear layer of CB-containing microparts, the electrical conductivity measurements suggested that the high structure CB has a greater tendency to form conductive pathways within the microparts via self-assembly even under high shearing conditions of μIM. However, no obvious synergistic effect of hybrid fillers on the construction of conductive pathways is detected, which is thought to be crucial to the enhancement of electrical conductivity for conductive polymer composites [11]. In addition to the weight ratios of CNT to CB in PS, further studies regarding the effect of intrinsic properties of polymer matrices on hybrid carbon fillers (i.e., CNT/CB) loaded polymer composites might be helpful to elucidate this phenomenon. Furthermore, the influence of annealing treatment on the electrical and morphological properties of as-molded microparts which have identical total filler concentration, i.e., 10 wt %, at various weight ratio of CNT/CB combinations was investigated as well. Results revealed that the electrical conductivity for CNT-containing samples increased after the thermal treatment whereas corresponding values for only CB-containing counterparts decreased. In this scenario, the occurrence of secondary agglomeration of CNT and the formation of CB aggregates are thought to be contributing factors. For example, a flow-induced orientation of CNT in microparts is expected due to the combined effects of high shearing and cooling in μIM. Thus, the reorganization of orientated CNT and the occurrence of secondary agglomeration of CNT facilitate the construction of conductive pathways. Besides, the formation of CB aggregates further promotes the formation of conductive pathways within the host polymer matrix. However, the aggregation of CB particles in only CB-containing samples might break down the continuum of conductive pathways, thereby leading to a reduction of electrical conductivity after the thermal treatment.

## Figures and Tables

**Table 1:** The ratio of (FD/TD)_σ_ for both the thick and middle sections of CB filled PS microparts.

Sample	(FD/TD)_σ_	
	Thick Section	Middle Section
PS/CB 3 wt %	12.6	N/A
PS/CB 5 wt %	5.6	4.3 × 10^5^
PS/CB 10 wt %	3.2	527

**Table 2:** Surface tension of pure components and the interfacial tension of PS-filler at 260 °C.

	*γ*	$\;\gamma_{i}^{d}\;$	$\;\gamma_{i}^{p}\;$	$\;\gamma_{12}\;$
	mN/m	mN/m	mN/m	mN/m
PS ^a)^	23.44	23.272	0.168	
CB ^b)^	21.77	19.59	2.18	
CNT ^c)^	27.8	17.6	10.2	
PS/CB				3.29
PS/CNT				11.82

^a)^ From reference [37]; ^b)^ From reference [34]; ^c)^ From reference [38].

**Table 3:** The average σ for the thick section of 10 wt % carbon fillers loaded PS microparts.

Sample ID	Thick Section-TD (×10^−^^5^ S/cm)	Thick Section-FD (×10^−^^5^ S/cm)
PS/CNT 10 wt %	6.59	127
10 wt %-PS/CNT70/CB30	20.2	155
10 wt %-PS/CNT50/CB50	14.1	137
10 wt %-PS/CNT30/CB70	28.2	172
PS/CB 10 wt %	62.9	201
